# Utilizing a novel high-resolution malaria dataset for climate-informed predictions with a deep learning transformer model

**DOI:** 10.1038/s41598-023-50176-3

**Published:** 2023-12-28

**Authors:** Micheal T. Pillay, Noboru Minakawa, Yoonhee Kim, Nyakallo Kgalane, Jayanthi V. Ratnam, Swadhin K. Behera, Masahiro Hashizume, Neville Sweijd

**Affiliations:** 1 Department of Vector Ecology and Environment, Institute of Tropical Medicine (NEKKEN), Nagasaki University, 1-12-4, Sakamoto, Nagasaki City, 852-8523 Japan; 2 Graduate School of Biomedical Sciences, Nagasaki University, Nagasaki City, Japan; 3 Department of Global Environmental Health, Graduate School of Medicine, The University of Tokyo: The University of Tokyo, 7-3-1 Hongo, Bunkyo Ward, Tokyo, 113-8654 Japan; 4 Limpopo Department of Health, Malaria Control: 18 College Street, Polokwane, 0700 South Africa; 5 Application Laboratory, Japan Agency for Marine-Earth Science and Technology (JAMSTEC), 3173-25, Showa-Machi, Kanazawa-Ku, Yokohama-City, Kanagawa 236-0001 Japan; 6 Graduate School of Medicine Department of Global Health Policy, The University of Tokyo: The University of Tokyo, 7-3-1 Hongo, Bunkyo Ward, Tokyo, 113-8654 Japan; 7 Alliance for Collaboration on Climate & Earth Systems Science (ACCESS), CSIR, Lower Hope Road, Rosebank, 770, Cape Town, South Africa

**Keywords:** Climate sciences, Malaria, Computational biology and bioinformatics, Computational models, Machine learning

## Abstract

Climatic factors influence malaria transmission via the effect on the Anopheles vector and Plasmodium parasite. Modelling and understanding the complex effects that climate has on malaria incidence can enable important early warning capabilities. Deep learning applications across fields are proving valuable, however the field of epidemiological forecasting is still in its infancy with a lack of applied deep learning studies for malaria in southern Africa which leverage quality datasets. Using a novel high resolution malaria incidence dataset containing 23 years of daily data from 1998 to 2021, a statistical model and XGBOOST machine learning model were compared to a deep learning Transformer model by assessing the accuracy of their numerical predictions. A novel loss function, used to account for the variable nature of the data yielded performance around + 20% compared to the standard MSE loss. When numerical predictions were converted to alert thresholds to mimic use in a real-world setting, the Transformer’s performance of 80% according to AUROC was 20–40% higher than the statistical and XGBOOST models and it had the highest overall accuracy of 98%. The Transformer performed consistently with increased accuracy as more climate variables were used, indicating further potential for this prediction framework to predict malaria incidence at a daily level using climate data for southern Africa.

## Introduction

The incidence of infectious vector borne diseases such as malaria are related to high mortality rates, with a high persistence in tropical and sub-tropical regions and a higher disease burden observed in the African continent. With the progression of climate change, increased variability and continued environmental modifications brought about by human activities^1–3^, the potential for changes in malaria transmission dynamics exists^4,5^. The World Health Organization (WHO) has set the goal of global malaria elimination, however there are still regions which have observed upward trends in cases, with a slowing rate of reduction since 2014^6,7^. The global technical strategy now includes malaria surveillance as a core intervention, especially since most malaria endemic countries have weak surveillance systems^7^. Consequently, malaria prediction frameworks can provide much needed tools and data to build and strengthen surveillance systems in these countries^8^.

Main sources of malaria predictions are provided by statistical^9^, and conventional machine learning models^10,11,35–37^. The accuracy can vary from 70 to 90% (Table 1), however, temporal resolutions for the predictions tend to be monthly or yearly^12^, with only some models able to provide weekly predictions^9^, while other studies only do classification analysis resulting in more qualitative outputs^35,37^. Despite statistical and machine learning models’ ability to provide relatively accurate predictions for malaria incidence, they have disadvantages. Statistical models for instance have short prediction windows and low temporal resolutions^14^. Kim et al^9^. limit forecasts to 16 weeks ahead to maintain prediction accuracy, which tends to drop in the second half of the 16-week window. When classical machine learning models have been used to forecast vector disease outbreaks such as weekly Dengue incidence^13^, it was found that deep learning model architectures tended to outperform the classical machine learning random forest models used (Table 1). However, the highest accuracy machine learning models already being used for malaria incidence prediction also tend to predict at a monthly or annual timescale^10,12^. This is due to data quality and resolution limitations^12^. Specifically, XGBOOST machine learning models^15^ have indicated the best prediction accuracy and efficacy compared to other machine learning methods used for malaria prediction. These predictions may be useful for longer term decision making, however may not be robust enough for managing and reacting to sudden outbreak events instigated by non-seasonal climate variability, therefore decreasing their ability to act as early warning systems throughout the year or for specific periods^15^.

**Table 1:** Notable past studies using machine learning and deep learning frameworks with climate data to predict malaria incidence or classification based outcomes.

Model	Accuracy	Data		Author/Date
Neural networks (MLP)	72.8	Monthly malaria data (1994–1999)	Temperature, rainfall, relative humidity, NDVI	Kiang et al. (2006)
Multiple classifiers + deep learning	70.3	Annual malaria incidence (2000–2017)	Rainfall, Temperature	Masinde (2020)
Artificial neural networks	82	Monthly malaria data (1995–2014)	Temperature, Rainfall, Relative Humidity, NDVI	Santosh & Ramesh (2019)
WEKA ML Tool and MLP	71	Monthly malaria data	Temperature, RH, Rainfall,	Mohapatra et al. (2020)
ML classification algorithms	25–93	2005–2011	Temperature, RH	Kalipe et al. (2019)
ML classification algorithms	80	1998–2020	Sea surface temperature variability (ENSO, IOD)	Martineau et al. (2022)

Recently, Transformer deep learning models have indicated strong performance for timeseries forecasting^43–46^. While not applied in malaria prediction studies, Transformer models have been applied to monthly dengue data or influenza prevalence and indicate better performance compared to other well-known deep learning models^38^. These Transformer class models have been successfully applied to influenza data^39^ and a series of other use cases as summarized by Ahmed et al^43^. Meanwhile, deep learning Transformer models have also indicated good performance on varying temporal resolutions from monthly, daily and even hourly timeseries datasets^40,42,43^. With the advancements in the field of deep learning, the ability for detection of complex relationships and patterns in the data has become easier^21^. Since deep learning models can learn and retain the relationships between predictor variables and the outcome (predicted variable), they can also be applied to other use cases and learn from large amounts of data dynamically by using all the data without requiring assumptions of error distribution or linearity^22,44,45^. This can allow deep learning models to predict larger timesteps compared to statistical or conventional ML. The ability for deep learning models to retain a memory of the relationships in the data and specifically in a timeseries, also allows for effective prediction for large complex multivariate timeseries data^23^.

Existing models use climate factors including but not limited to rainfall, temperature, relative humidity and NDVI as dependent variables in the model construction (Table 1). Some of the first studies^1,16^ which considered incorporating climate data for malaria prediction indicate the high levels of accuracy of these climate driven models when temperature and precipitation were used. Climatic variables and climate-based indices such as the Indian Ocean Dipole have also been shown to influence malaria case incidence and timing^17,35^. As a result, the use of these climate variables which also have robust dataset availability, can be applied conveniently to malaria prediction problems for most countries^43^. However, the relationships between climate variables and malaria incidence are not always linear, presenting a challenge to capture the complexity of interactions between rainfall, entomological factors and malaria incidence when building statistical models^18,19^. The complex mechanistic models which do incorporate complex biological and epidemiological factors are usually used at a global scale and are not specific enough to provide high spatial or temporal resolution predictions that can inform on-the ground interventions for specific areas^18,20^. The advantage of deep learning Transformer models can leverage high granularity data to understand the subtle relationships between climate and malaria incidence more accurately than if lower resolution data were used^43,44,48^.

Most existing use cases of deep learning models for vector-borne diseases feature studies on Dengue and do not explore the use of Transformers^24,38^. Ho et al^25^. utilized machine learning approaches to identify laboratory confirmed Dengue cases but used epidemiological factors instead of climate-based predictors, indicating the flexibility of deep learning models. Deep learning models have also been leveraged to predict malaria in China^26,47^, however, there has been no application of equivalent models in Africa. While established deep learning models such as Long-Short-term Models (LSTMs), Recurrent Neural Nets (RNNs) or Generative Adversarial Networks (GANs) exist for timeseries prediction, they usually struggle to predict long time sequences with complex temporal dependencies^12^. The existing sequence to sequence models (take inputs and create an output sequence) have difficulty retaining the first elements from the data sequence^21,27,47^. Very few studies examine malaria in southern Africa with deep learning methods (summarized in Nkiruka et al^15^. and Mbunge et al^37^.). Martineau et al^35^. uses Sea surface temperature variability and classical machine learning to predict outbreak classifications at a monthly resolution. When daily malaria data was used in South Africa, it was not done with a deep learning framework but a SARIMA model (Adeola et al^36^.). Overall, there is a lack of studies that use a generative prediction deep learning model with high-resolution malaria and climate data. These shortfalls can be accommodated by creating a predictive framework to add knowledge regarding the efficacy of pairing high resolution malaria data with a state-of-the-art modified Transformer deep learning model in the current malaria prediction domain of Southern Africa.

This paper focused on the relatively new deep learning model architecture known as the Transformer with attention. The use of Transformers in malaria incidence predictions for a country in Africa (which accounts for over 95% of cases worldwide^7^) has not been explored and is an addition to the existing studies in Africa which have mainly used lower resolution malaria data with classical machine learning models^15,35^. Transformers take a different approach to timeseries prediction and are capable of capturing and retaining long term dependencies in the data and can be useful when using complex climate and health data^27^. The aim of this study was to compare the prediction accuracy and robustness between an existing statistical model adapted from Kim et al^9^., the current gold-standard machine learning XGBOOST model^15^, and a deep learning model using Transformers to determine wether the Transformer is viable as a long-term solution for malaria prediction. The end goal of this study was to improve on conventional deep learning models and substitute or complement existing statistical and machine learning frameworks such as the aforementioned XGBOOST models^15^, to enable reliable, generaliseable and consistent predictions of disease influenced by climate factors at different temporal resolutions.

In contrast to existing literature^37,38^, which largely focuses on traditional endemic regions and uses lower temporal resolution data for malaria or other vector borne disease prediction, this study introduced multiple additions to the forecasting of malaria with deep learning. Firstly, the dataset originates from the province of Limpopo in South Africa, a region that is not typically endemic for malaria but experiences sporadic outbreaks, predominantly from imported cases related to neighboring regions such as Mozambique^9^. This geographic focus lends a unique context for malaria prediction using climate data. Secondly, the high-temporal-resolution dataset, collected daily, stands as a rarity in health-related malaria data due to the challenges in gathering and maintaining such datasets in affected countries of Africa due to economic and social challenges^43,47^. The high granularity of this data allows us to train more accurate and robust predictive models^44^, thereby offering a significant methodological advance over prior work that often relies on monthly data which cannot capture daily climate signals that may be present in the malaria forecasting environment^44^. In addition to using traditional climate data, this model incorporated future climate projections from the JAMSTEC global climate models, enhancing the realism and applicability of our forecast test scenarios. Finally, a novel loss function was specifically tailored to the unique characteristics of our dataset, further optimizing the Transformer model's predictive capabilities. Collectively, these factors not only reinforce the importance of the malaria data but also underscore the methodological innovations introduced in this study and places the study in a position to contribute to the understanding of deep learning Transformers and their applicability on high resolution malaria data in Southern Africa.

## Methodology

### Malaria surveillance data

Malaria case data was acquired for Limpopo province located in South Africa from 1998 to 2021 from the Limpopo Department of Health Malaria Program. The malaria cases recorded by the health departments system are based on positive blood smear results or malaria rapid diagnostic tests. Only the case count data and local or non-local case status metrics were extracted from the database and used in this study following Kim et al^9^. Case data were extracted and compiled into daily counts over the extraction period. The data was completely anonymous.

### Observational climate data

To compare the DL model to the statistical model, precipitation and temperature were used following Kim et al^9^. The data were extracted at a daily scale from National Oceanic and Atmospheric Administration (NOAA)/National Center for Environmental Prediction (NCEP) from the NCEP-DOE Reanalysis II dataset^35^. The precipitation and temperature were extracted and averaged over the study area of Limpopo province (22·3° S to 23·0° S and 29·2° E to 3 0·6° E). Additional climate variables were extracted and averaged for the same study area coordinates to test the deep learning model on multiple new variables, which included evaporation, near surface windspeed and indices such as the Indian Ocean Dipole, Southern Annular Mode and the Niño 4 index. The statistical and XGBOOST were not tested with additional climate variables as this was done in Nkiruka et al^15^. and the statistical model does not perform well with too many additional variables^9^.

### Data and modelling workflow

**Figure 1:**
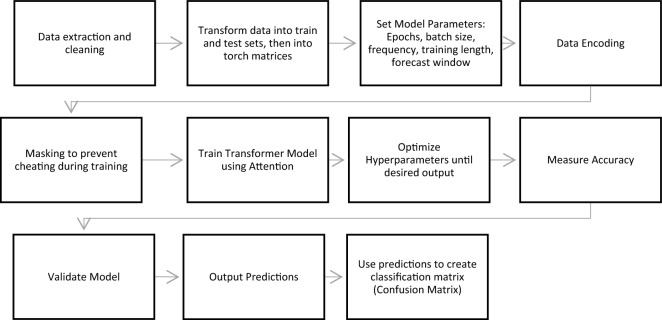
Workflow and progression indicating the processes applied to the data and the training and result evaluation.

The processed malaria case data and climate data were combined and separated temporally into train (1998–2020) and test (2021) sets. For the statistical model, the daily case, temperature, and rainfall data were aggregated to a weekly level. The XGBOOST and Transformer models were provided with daily data for training. Weekly Transformer models and XGBOOST models and a daily Statistical model (Appendix 5) were also tested but excluded due to low performance. The aim was to test and evaluate each model in their best performance range. The model parameters for the Transformer, including, Epochs, batch size, frequency, training length and forecast window (See Appendix 2) were tested and adjusted until the best possible training results and prediction accuracy were attained (Fig. 1). In the Transformer, the loss function was used to quantify the discrepancy between the model's forecasts and the actual data, guiding the optimization of the model parameters to improve predictive accuracy over iterations (Appendix 2). The existing loss functions (MSE and smooth loss^56^) were tested along with the novel loss function developed for this study’s specific prediction framework. The statistical model was trained on the weekly malaria and climate data using the same methodology as outlined in Kim et al^9^. Following the training phase, the three models were used to predict up to 2 years of malaria cases. The raw numerical predictions were used in conjunction with the ground truth or actual case count data to assess and evaluate the model’s using classification and regression metrics of accuracy.

### Accuracy evaluation: threat level thresholds

Once both model’s predictions were attained in the form of weekly (statistical) and daily (XGBOOST and Transformer) malaria count data, the prediction output data were converted into classes. Specifically, classified into low, medium and high malaria case groupings based on 30th, 60th and 90th percentiles respectively. The percentiles (See appendices) were chosen based on threat thresholds for historical malaria cases during the South African malaria season (September to May) over the 1998–2021 period following Kim et al^9^. and Teklehaimanot et al^48^. By creating threat thresholds, the assessment of the model in a real-world setting can be tested, as thresholds alerts levels can allow for detection of when the disease may increase to a higher risk level^49^. Following the classification of the case count data into the three classes, confusion matrices were applied to make a comparison between the statistical and deep learning model performance in predictions. Other measures of accuracy were also compared, including sensitivity, specificity, prevalence, balanced accuracy, negative and positive prediction values^9^. To assess classification accuracy, metrics including F1, f-beta and kappa scores, precision and recall were calculated^28^. The Area Under the Receiver Operating Characteristic (AUROC) was used to assess the classification accuracy of the models, while the Area Under the Precision-Recall Curve (AUPRC) was employed to evaluate the precision-recall tradeoff, especially in the context of imbalanced datasets^42^. The one-vs-all approach is used for the AUROC and AUPRC, where a class is compared against all other classes.

### Accuracy evaluation: regression analysis

The numerical case predictions from the models were used to compute multiple regression-based metrics to assess the performance of the models’ actual malaria case predictions. Explained variance, max error, MAE and R^2^ metrics specifically were calculated for each model^50^. These regression metrics were used to evaluate the numerical prediction output of malaria cases from each model to assess the performance of the models’ predictions in relation to the ground truth values of malaria cases^50^.

### Transformer model architecture

The Transformer with attention model used to process and predict on the timeseries data was adapted from the original created for sequence-to-sequence predictions in Vaswani et al^21^. Transformers are a newer model architecture which relies on an attention mechanism which can maintain a memory of dependencies between predictors (inputs) and predictions (outputs), replacing the recurrent models usually used for sequence data such as timeseries and which are unable to maintain a memory for larger datasets^21^. A detailed mathematical definition of the Transformer is presented in Thickstun^29^. The actual model architecture was adapted from Vaswani et al^21^. Our study employs a decoder-only Transformer architecture, optimized for the task of time-series prediction. The choice of using only the decoder component is motivated by its efficiency and suitability for generative tasks, as validated by prior studies in the field^43,51^. Furthermore, we introduce a novel loss function tailored for our high-resolution malaria dataset, enhancing the model’s predictive capabilities.

**Figure 2:**
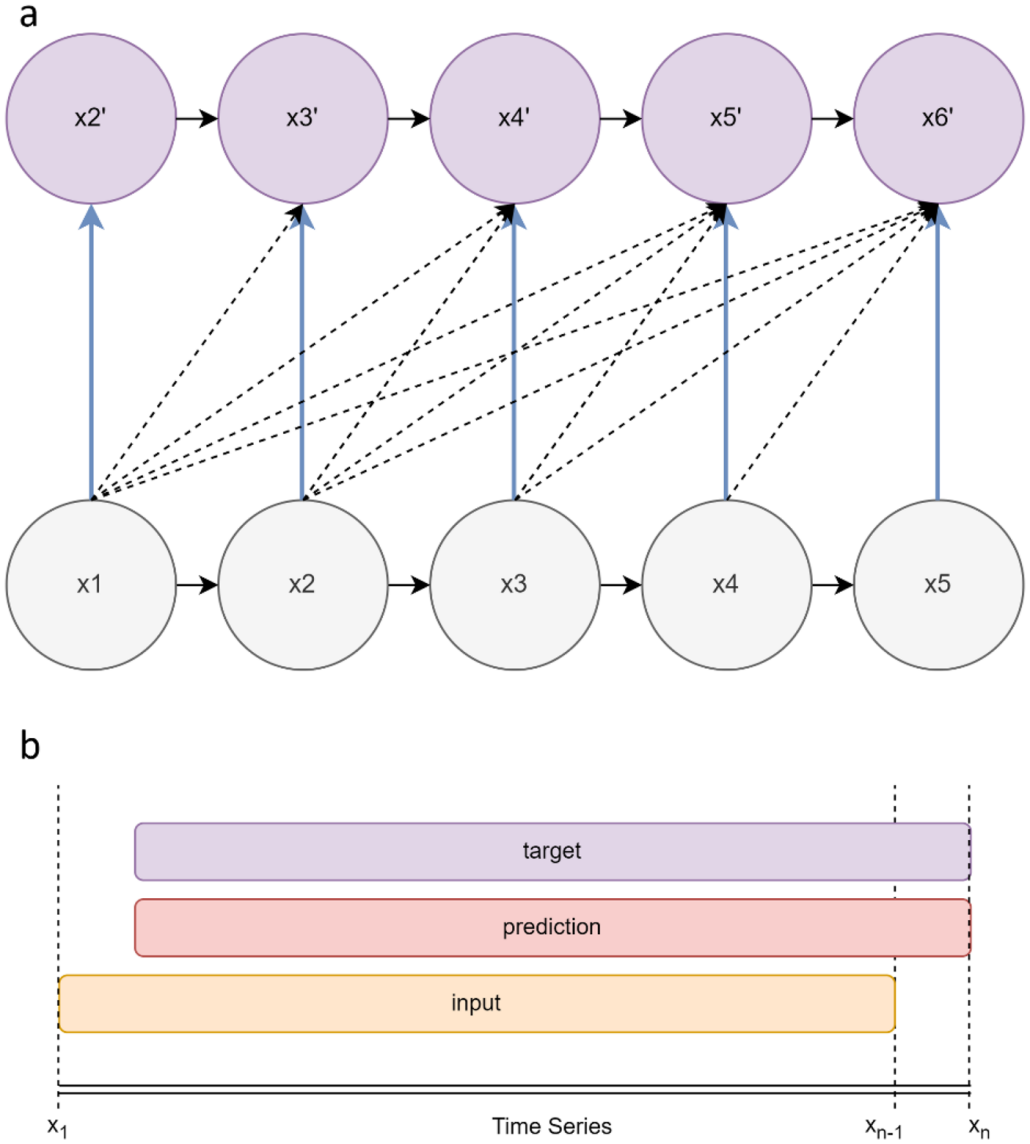
(**a**) Representation of self-attention. Connections are maintained throughout sequence as model trains. The purple circles indicate the predictions, and the dotted arrows are the attention mechanism keeping all information connected between predictions so that downline future predictions still retain and have access to the information in the earlier predictions. The model predicts × 2′ for the first input of data, uses the predicted data to predict the next value × 3′. After all predictions are made, the loss is calculated between actual input (× 1… × 5) and predicted outputs (× 2′… × 6′). (**b**) The input is the malaria timeseries and climate data, the target is the sequence shifted to the right by one time step so for each new input, the model will output a prediction.

The Transformer-decoder setup is used for predicting future values in a time series based on the provided past values. Firstly, an input sequence of data points from a time series is fed into the model, for example, a sequence of length 5 denoted as × 1, × 2, × 3, × 4, × 5 (Fig. 2). The model attempts to predict a target sequence which is the input sequence shifted one step to the right, denoted as × 2, × 3, × 4, × 5, × 6. The prediction process unfolds step-by-step. Firstly, with only × 1 available, the model attempts to predict × 2, denoted as × 2′. In the next step, having the true values × 1 and × 2, it predicts × 3, denoted as × 3′, and this pattern continues. In each step, the model receives all true values available up to that point to make the next prediction. The model’s output is a sequence of these predicted values: × 2′, × 3′, × 4′, × 5′, × 6′. During training, this predicted sequence is compared to the true target sequence to calculate the loss, which is then used to update the model’s parameters. Each prediction in the sequence contributes equally to the total loss, aiding the model in learning and refining its predictions for better accuracy in subsequent iterations.

### Self-attention mechanism

The self-attention function involves the input which needs to be represented by 3 dimensions (query, keys, values) which are mapped to an output. These three factors are data abstractions used in the attention modules calculations and are derived by multiplying inputs by three weights (Eq. 1). This can be done multiple times for each input (multi-attention head mechanism) allowing for precise association control between malaria cases and climate variables. The attention mechanism facilitates a focus on the most important or relevant input vectors (self) while calculating the output vectors (prediction). This aids the model to focus less strongly on irrelevant features in the data. The attention mechanism works in three main ways during model training, which allows the mechanism to determine many different probable predictions based on the results calculated at different stages of the model’s architecture (Appendix 1), basically allowing it to draw information or dependencies from the different inputs and hidden states at any point in the timeseries (Fig. 2). While the model attempts to determine the relationships between climate states and malaria incidence, the self-attention module helps the model associate the specific magnitudes of each climate variable with the most probable malaria case count outcome.

$$Attention(Q,K,V)=softmax\left(\frac{Q{K}^{T}}{\sqrt{{d}_{k}}}\right)V$$

Optimized matrix operation used in attention-head mechanism during training.

1 $$\begin{gathered} X_{n} \times W_{n} Q = Q_{n} \hfill \\ X_{n} \times W_{n} K = K_{n} \hfill \\ X_{n} \times W_{n} V = V_{n} \hfill \\ \end{gathered}$$

Creating the Query(Q), Key(K) and Value(V) vectors for each input. During training the model learns these three weighted matrices after multiplying them by the input (X).

2 $${Z}_{n}=softmax\left(\frac{Q{K}^{T}}{\sqrt{{d}_{k}}}\right)V$$

The self-attention score is the dot product between Q and the K vector to determine how relevant each K is to the current Q. The score is calculated to determine the importance of inputs in relation to all other inputs in the timeseries sequence. Higher scores indicate higher relevancy. The resulting score matrices (Z_n_) are then passed to the softmax function to ensure it is positive and adds to one.

3 $${Z}_{0}+{Z}_{1}+\dots {Z}_{n}$$

Since there are multiple attention heads, there are multiple resulting Z matrices calculated for each input. These are then concatenated.

4 $$Output\, layer{=Z}_{n}\times {W}^{o}$$

The concatenated Z matrices are multiplied by a Weighted matrix (W^o^) to produce an output layer which is then sent through the model to be decoded and presented as an output.

### Scheduled sampling

Scheduled sampling adopted from Bengio et al.^30^ was used to help the model correct its mistakes during training. This sampling method first feeds the model true values to correct its errors, then as the training progresses, the model is fed its previously generated predictions instead of the true value (See Appendix 1 for full details). The sigmoid decay function^30^ was used in this study to facilitate the sampling change over time. This sampling was used to prevent overfitting and promote generalization and robust modelling. In addition, the model was instantiated with a dropout of 0.2 (Appendix 1), allowing for optimal prediction without overfitting^40^.

### Novel loss function

Loss functions in deep learning measure the discrepancy between the model's predictions and the actual data^56^. They are the objective for optimization algorithms, guiding them to adjust the model's parameters to minimize this discrepancy. By minimizing the loss function during training, the model learns to make more accurate predictions, leading to better performance in predictions. A new loss function (Eq. 5) was created specifically for variable case incidence datasets. The methodology we used entailed taking aspects of the MAE (Mean Absolute Error), MSE (Mean Squared Error) and Huber loss functions and creating a more adaptable loss function to suit real-world timeseries data. From here we will refer to our novel function as an M-Delta function. The M-Delta performs similarly to the Huber loss function which behaves similarly to the MSE for small errors and the MAE when larger errors between predictions and actual observations occur. The M-Delta specifically uses an adaptable delta threshold hyperparameter. This allows the model to choose which loss calculation (MAE or MSE) to transition to when assessing the predicted values against the true values depending on the delta. For small errors which are <  = delta, the MSE function is used and will penalize large discrepancies between the predicted and actual values. However, when the delta is exceeded due to very large discrepancies which usually indicate malaria outbreak periods, the loss function will become linear (similar to MAE). This was important to decrease sensitivity to outlier events at times such as outbreaks. The adaptability of this function allows it to change based on the delta which is influenced and determined by the distribution of the data, so instead of a single delta for the whole dataset, a unique delta is computed for each batch of data during training based on the batch’s standard deviation. This was implemented in python, but the mathematical notation is provided for understanding.

- *y*_true,*i*​_: true value for *i*-th instance per batch.
- *y*_pred,*i*​_: predicted value for *i*-th instance per batch.
- *σ*: standard deviation for *y*_true_​ per batch.
- *N*: number of instances in the batch.

5 $$L\left( {y_{{{\text{true}}}} ,y_{{{\text{pred}}}} } \right) = \frac{1}{N}\sum\nolimits_{{i = 1}}^{N} {\left\{ {\begin{array}{*{20}l} {\frac{1}{2}\left( {y_{{{\text{true}},i}} - y_{{{\text{pred}},i}} } \right)^{2} } \hfill & {if\,\left| {y_{{{\text{true}},i}} - y_{{{\text{pred}},i}} } \right| \le \sigma } \hfill \\ {\left| {y_{{{\text{true}},i}} - y_{{{\text{pred}},i}} } \right| - \frac{1}{2}\sigma ^{2} } \hfill & {{\text{otherwise}}} \hfill \\ \end{array} } \right.}$$

### XGBOOST model

An eXtreme gradient boosting model (XGBOOST) is a supervised machine learning method used to model regression or classification problems and has shown promising results in the malaria climate prediction field^15^. Comparing a statistical model to a deep learning model has fundamental challenges, therefore in order to highlight the strengths of the Transformer model in this paper accurately, an XGBOOST model was also compared to the deep learning framework. The XGBOOST was used due to it outperforming other classical machine learning algorithms in the prediction of malaria incidence^15^.

## Results

**Figure 3:**
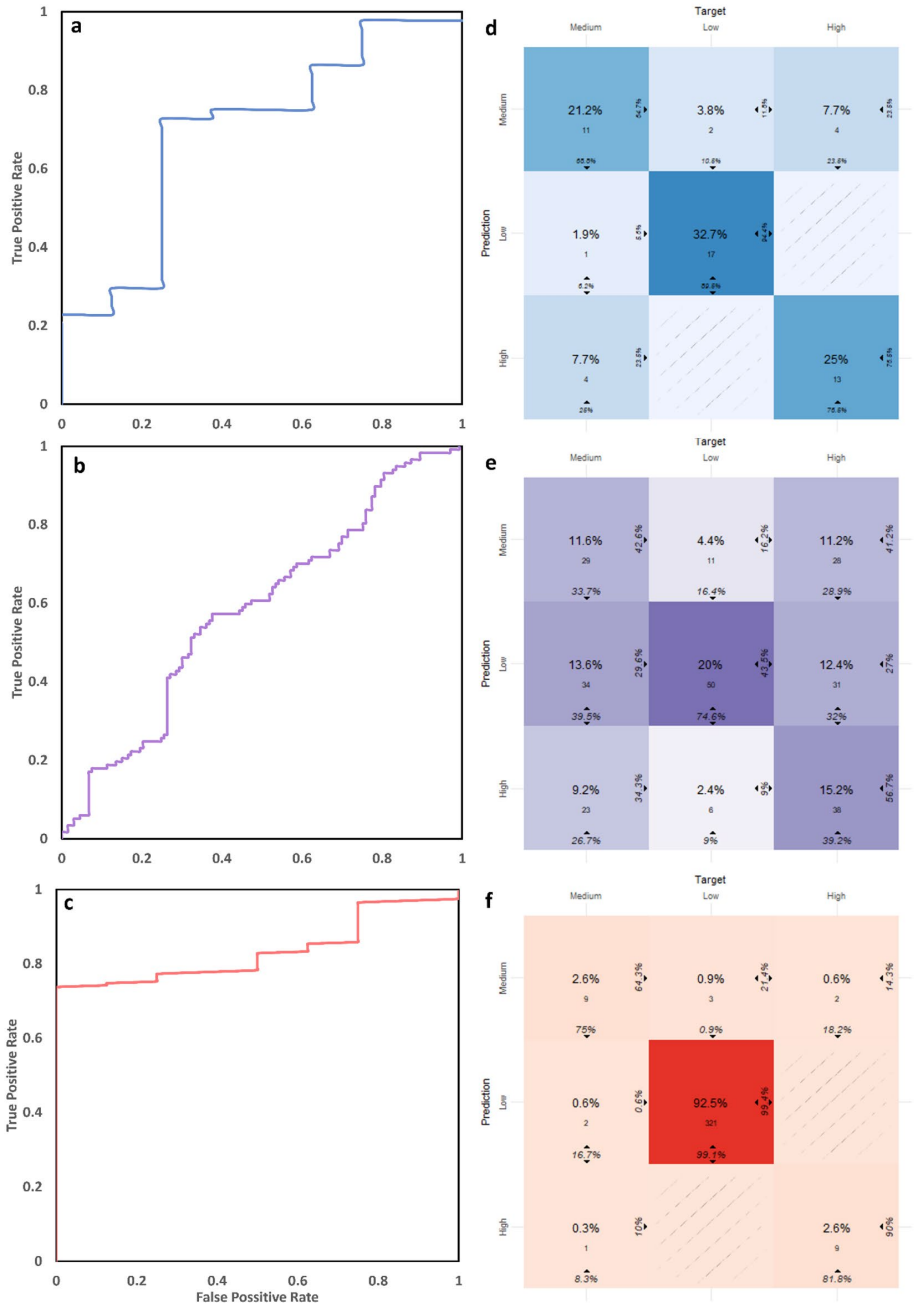
The area under receiver operating characteristic (AUROC) curves for the statistical model (**a**), the XGBOOST model (**b**) and the Deep learning transformer (**c**). The curves indicate the probability of the model predictions being correct at various thresholds. The ROC curves and AUC indicate probability of a model attaining a true prediction. The closer the curve is to the top left of the graph, the better. Confusion matrices for the statistical model (**d**), XGBOOST model (**e**), and the Deep learning transformer (**f**). The matrices indicate the normalized count/overall % prediction accuracy in the middle of each tile. The bottom and side values indicate the percentage of correct classifications of the target (High, Medium, and Low) in the column and row respectively.

Using the statistical and deep learning models raw numerical predictions, classes of low, medium, and high malaria case incidence were derived (Table2). The prediction accuracy metrics were then computed based on how accurately the models matched the actual classes. The statistical model had an overall accuracy 78·8% (F1 = 0·64). When evaluating the performance of the model’s prediction with AUC (Fig. 3a–c), the scores of 0·43 for the statistical model with no malaria case predictor and 0·69 when it was used to evaluate 2021 malaria cases only (Table2) are clearly lower than the Transformer. The XGBOOST performance is low, with an AUC of 0·53. The Transformer model however indicates a higher accuracy of 98% and an AUC of 0·83 with the highest observed F1 score of 0·8 for daily predictions. The AUC, F1 and Kappa scores indicate that the probability of correctly predicting a malaria case class is very low for the XGBOOST machine learning models and the statistical models (Tables 2 and 3). Kappa values are highest for the Transformer indicating high agreement between predictions and actual cases. The statistical model has a moderate kappa value of 0·68, while the XGBOOST value is close to zero indicating the weakest agreement between actual and predicted case classes. The confusion matrices for the statistical model (Fig. 3d) indicate 94·4% accuracy in identifying the “low” malaria case class correctly, while the Transformer (Fig. 3f) has an accuracy of 99·4% at a daily level. The statistical model has lower accuracy in predicting medium case incidence classes, only attaining 64·7% accuracy at predicting these classes. This is also observed for the Transformer model which identified medium intensity malaria cases for 2021, 64% of the time. The “high” class prediction had an accuracy of 76·5% for the statistical model but 90% for the Transformer. The XGBOOST models failed to attain an accuracy level above 60% for any of the classes (Table 2).

**Table 2:** Summary of model accuracy for statistical, XGBOOST and deep learning transformer.

Model	Sample no	Overall accuracy (%)	Low (%)	Medium (%)	High (%)	AUROC	AUPRC	*p*-value	F1
XGBOOST classification (D)	n = 702d	46.8	57.7	32.5	38.9	0.5367	0.3425	0.007	0.4631
Statistical 0 biased (2021)	n = 52w	78.9	94.4	64.7	76.5	0.70	0.3490	< 0.001	0.6428
Statistical (2020–2021 no mal predictor)	n = 104w	54.8	71.4	41.2	51.4	0.43	0.3760	< 0.001	0.3548
DL Transformer (D, 2021)	n = 360d	97.7	99.4	54	90	0.83	0.4296	< 0.001	0.8472

Low, Medium, and High indicate accuracy % of predicting that class. Accuracy is a rounded metric of overall prediction accuracy calculated from true positives. The *p*-value provides a measure of statistical significance regarding the overall accuracy of the model. The F1 score reflects the model’s balance of precision and recall in predictions on the dataset, with 1 being a perfect score. The Statistical 0 biased label indicates that the model was allowed to use actual malaria data as a predictor during training and prediction.

**Table 3:** The classification and regression accuracy measures used for model evaluation.

	Statistical	Statistical (No mal)	XGBOOST	Transformer
Classification accuracy metrics				
f1	0.6429	0.3548	0.3243	0.8472
fbeta	0.5965	0.3572	0.3250	0.8458
kappa	0.6824	0.3221	0.1453	0.8207
Regression accuracy metrics				
Explained variance	− 0.0605	− 1.7744	− 0.9173	0.8659
Max error	194.4000	728.9162	176.6754	40.9351
MAE	38.1327	136.7020	12.3079	8.1887
R2	− 0.9574	− 3.3725	− 1.0448	0.8413
*p*-value	< 0.001	< 0.001	0.057	< 0.001

The F-beta score indicates the model’s balance between precision and recall in predictions on the dataset, with a beta value of 0.5 placing more emphasis on precision. The kappa indicates the agreement of the model at predicting a class (low, medium, and high), with 1 being perfect agreement and below 0.4–0.6 as being moderate agreement. The regression accuracy metrics are calculated from the actual malaria cases predicted (not the classification results).

**Figure 4:**
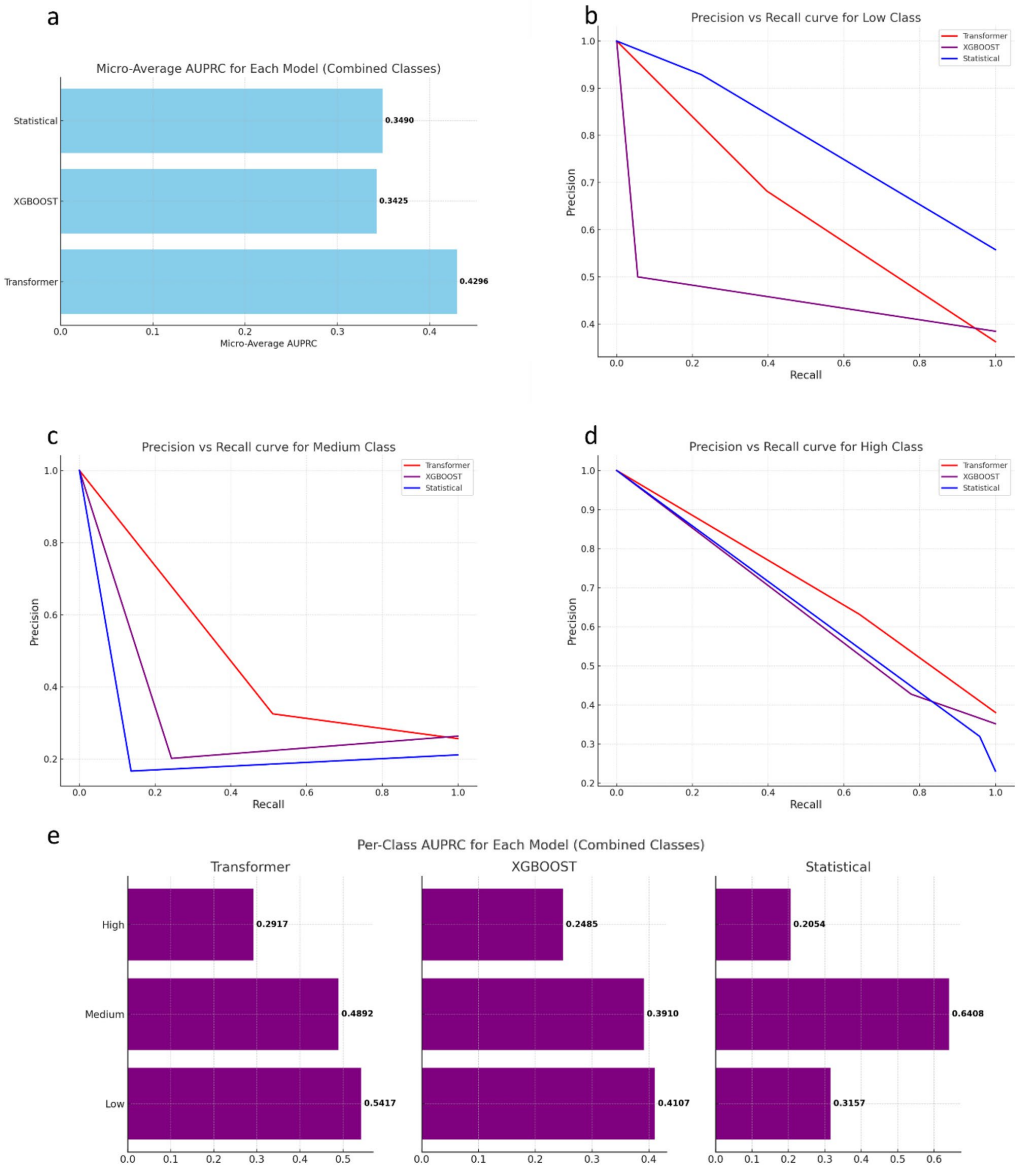
AUPRC calculated for the statistical, XGBOOST and Transformer models. (**a**) combined performance of all classes per model. Precision is the measure of correctly identified positive cases from all the cases predicted as positive. Recall is the measure of correctly identified positive cases from all the actual positive cases. Precision-Recall curves which demonstrate the low false positive rate desired when precision is high and low false negative rate when recall is high are calculated for (**b**) Low class, (**c**) Medium class and (**d**) High class. (**e**) Per class AUPRC performance for the Transformer, XGBOOST and Statistical model.

**Figure 5:**
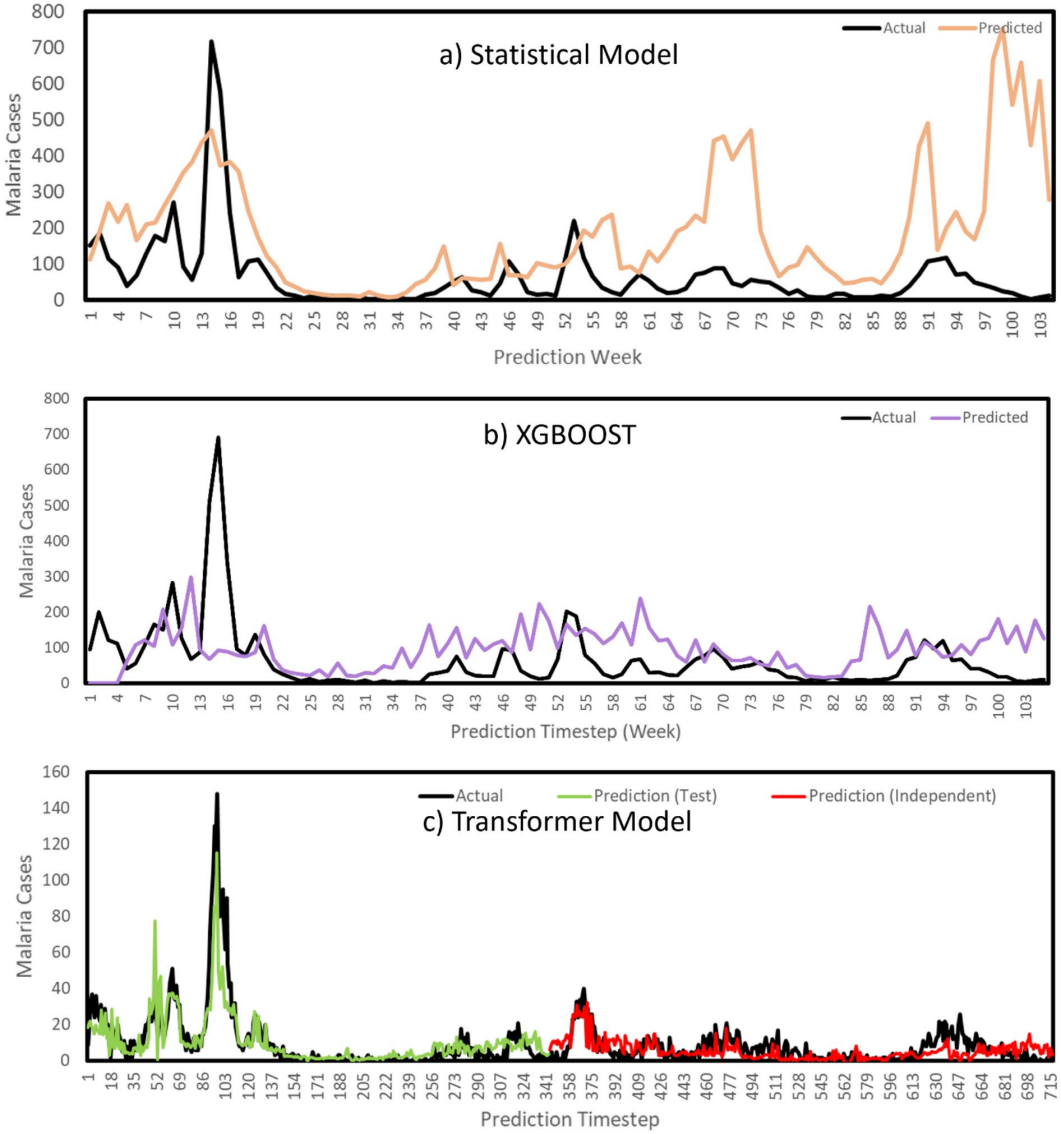
Malaria incidence predictions against actual malaria cases by model. Statistical model (**a**) and Deep learning transformer (**b**–**c**). (**a**) and (**b**) indicate predictions at a weekly level, while (**c**) is predicting at a daily level with all models covering a prediction period of approximately 2 years. The Black lines indicate the actual historical observations of malaria cases. The coloured lines indicate the respective model predictions. The green line for the Transformer (Panel c) indicates the models performance on the test set where inference was performed with the model looking one day ahead to make a prediction for the following day, but the model had the actual malaria cases masked to prevent looking to far ahead or cheating. The red line then indicates a pure prediction using only climate data to determine the case outcomes with the trained model using its own past malaria predictions to determine the consecutive daily predictions. See Appendix 5 for correlations between predicted and actual malaria values.

The performance of these models was also evaluated using the AUPRC for each class separately (Fig. 4e), as well as a micro-averaged AUPRC across all classes (Fig. 4a), to ensure minority classes were accounted for. For the high class (Fig. 4d), the Transformer model yielded the highest AUPRC of 0·2917, followed by XGBOOST with 0·2485, and the statistical model with 0·2054. In the medium class (Fig. 4b), the Statistical model outperformed with an AUPRC of 0·6408, with the Transformer and XGBOOST models attaining AUPRC values of 0·4892 and 0·3910 respectively. However, in the low class (Fig. 4b), the Transformer model achieved the highest AUPRC of 0·5417, surpassing the XGBOOST and statistical models which scored 0·4107 and 0·3157 respectively. In terms of micro-averaged AUPRC across all classes, the Transformer model again led with a score of 0·4296, while the statistical model exhibited a slightly better performance than XGBOOST with scores of 0·3490 and 0·3425 respectively. These results suggest a varying performance of the models across different risk classes, with the Transformer model demonstrating a relatively more consistent performance across the classes. In addition, the overall correlation between actual and predicted values (Fig. 5, Appendix 5) r = 0·859; R^2^ = 0.70 (*p* = 0.003) indicates the Transformer’s numerical predictions are also more consistent with higher accuracy. All models appeared to fall into the class imbalance problem due to the larger number of low case classifications, however only the transformer was able to accurately predict these low classes, showing strong performance as evidenced by the high values in the AUROC, AUPRC, F1, and Kappa metrics. The Transformer was able to maintain a higher true positive rate and correctly predicts outputs that map to the alert level classes with high accuracy.

Evaluating the model predictions using a regression framework indicated the prediction accuracy for actual daily malaria case numbers for the Transformer and weekly for the statistical and XGBOOST machine learning models. The Transformer had the best scores across all regression metrics tested (Table 3). The explained variance indicated the model accounted for 87% of variability in the dataset. The negative explained variance for the statistical and XGBOOST models supports their failure to predict higher case numbers when outbreaks actually occurred. Max error was lowest for the Transformer, compared to the other two models. This indicates a lower tendency to predict larger outbreaks when they may not actually occur. The R^2^ scores were highest for the Transformer (R^2^ = 0·84). Meanwhile the other two models had negative R^2^ scores, indicating that their predictions performed worse than a constant function (naïve model) that could always predict the mean of the data.

**Figure 6:**
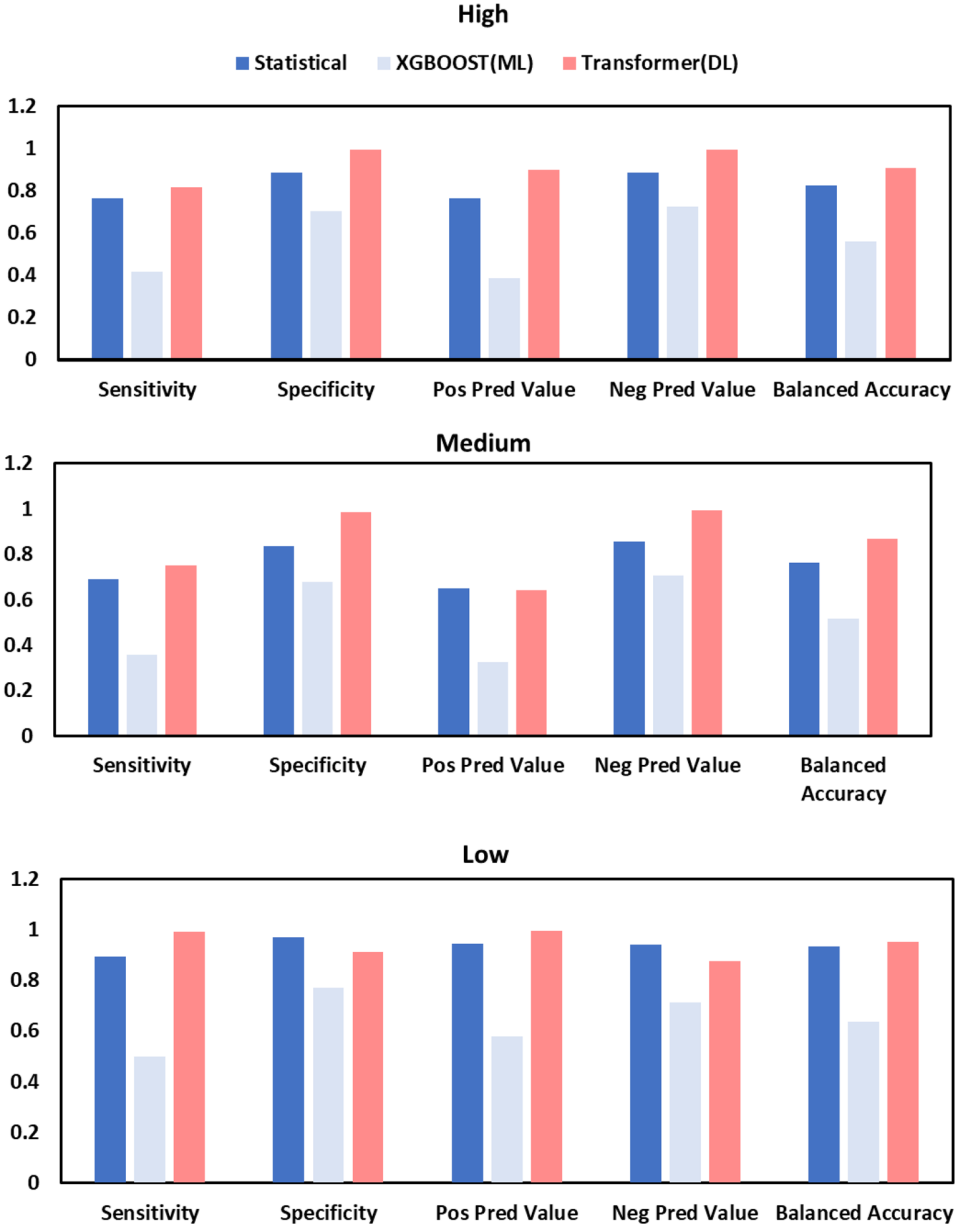
Classification accuracy metrics for High, Low and Medium classes of malaria incidence as predicted by the three models. Sensitivity is a measure of true positive classifications, while specificity is the measure of true negatives. The positive and negative predictive values indicate the probability of predicting a true positive or true negative out of all positive and negative cases respectively. The balanced accuracy is derived from the mean of sensitivity and specificity.

**Figure 7:**
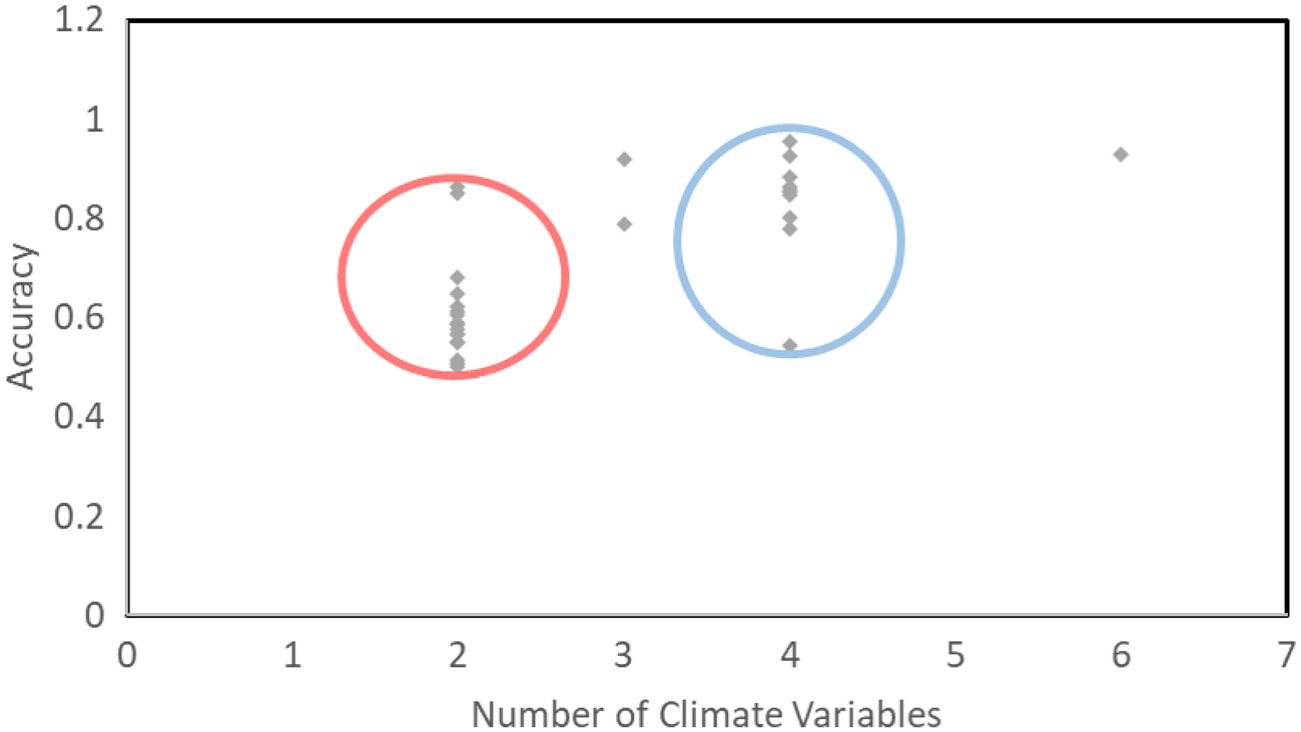
The number of climate variables used in the Deep learning transformer during training and the accuracy attained during prediction. The red circle indicates only temperature and rainfall. The blue circle has multiple other climate variables including but not limited to relative humidity, evaporation rate, near surface windspeed etc. See Appendix 2 for detailed information.

The actual case number predictions (Fig. 5) indicate that the statistical model and the Transformer were able to predict very closely to the actual case numbers, however the statistical model was doing this at a weekly scale when compared to the deep learning models daily prediction scale. Notably, the Transformer performed best when using the novel M-DELTA loss function during training by more than 20% compared to the baseline MSE loss function (Appendix 2). In addition, when classifying the Transformer predictions at a weekly level, the classification matching rate was basically perfect for the 2021 prediction year. On balanced accuracy (Fig. 6) the Transformer also outperformed the other models at predicting all malaria case alert level classes. Furthermore, during the training of the Transformer, the use of additional climate variables with rainfall and temperature indicated that higher accuracies could be achieved consistently (Fig. 7, Appendix 2).

## Discussion

The statistical model and XGBOOST model have been used previously with climate data for malaria prediction and have been further evaluated in this study. The statistical model used here is a distributed lag nonlinear model adapted from Kim et al^9^. The model is currently able to provide good short-term predictions for the Limpopo area from 2 to 16 weeks ahead. However, the statistical model’s accuracy is misleadingly high as a result of the data presenting an imbalance problem, in this case a majority of predictions classified as low^28^. This satisfies the majority of cases in reality as a majority of the dataset either has zeroes or is classified as low case incidence and therefore does not necessarily indicate the modelsprediction ability. This is clearly seen once more robust statistics such as the AUROC and AUPRC were used, whereby the model fails to demonstrate predictive ability and has relatively low precision-recall performance (Figs. 3 and 4). The low performance of the statistical model to predict accurately except for medium alert levels found here can add to information of previous studies using similar models^9^. While the XGBOOST underperformed on weekly predictions with the Limpopo malaria dataset in this study, it has indicated high accuracy and AUROC scores when predicting malaria cases at a monthly scale and tends to outperform many other machine learning models on this task especially when using multiple climate variables^15^. This study only used temperature and rainfall, and the same level of data processing was not replicated as done in Nkiruka et al^15^.

This study focused on developing a deep learning Transformer model to predict malaria cases using a high-resolution novel malaria dataset with equally high resolution climate variables as predictors. When comparing the deep learning model to existing statistical and XGBOOST (machine learning) models^15^, it was apparent that the Transformer was able to predict malaria cases with higher fidelity and consistency according to both classification evaluation metrics (AUROC and AUPRC) and regression accuracy metrics (explained variance, MAE, R^2^). While the Transformer model output is numerical, it was convenient to convert the daily prediction results to a classification problem to compare it to the weekly data from the statistical model^42^. This allowed for a generalised comparison and accounts for the difference in nature of statistical and deep learning models and the temporal resolution^14^. The classification metrics used here to evaluate the model have also indicated better performance than existing machine learning classification techniques^35^. For instance, Mohapatra et al^31^. used a classification model based on monthly malaria and climate data, which underperformed compared to the Transformer, with a calculated kappa of 0.63, RMSE of 0.6 and accuracy of 0.71.

While the field of epidemiological forecasting is still in its infancy, there have been studies which leverage deep learning effectively, for instance Mussumeci and Coelho^13^ used a LSTM deep learning network to predict weekly Dengue cases and the pattern of predictions were similar to results observed here (Fig. 6), whereby the initial prediction period had very high accuracy, but once the model predicted on data not in the training or test sets (validation data) it tended to be less accurate compared to the earlier prediction periods. This is to be expected and even encouraged as it is better to have a model that can generalise, in order to avoid overfitting^32^. Generalizability promotes real-world deployment and potentially different regions and other climate-related health outcomes^39,43^. Despite this decrease in accuracy, it still maintains higher accuracy performance over a longer period, highlighting one of the advantages of using deep learning models such as the Transformer which can retain memory of the relationships between the predictors and the outcome across the dataset regardless of temporal resolution^21,22^. Multiple modelling studies^41–43,45–47^ leveraging a Transformer for timeseries prediction tasks have consistently found that these models can outperform established deep learning and machine learning models regardless of the complexities such as dataset size, temporal resolution^44^ or number of predictors^38,40^ or domain characteristics^49^. In the application of a high-resolution malaria dataset which presented many of the above complexities, the results indicate the Transformer predictive framework is effective for providing data that can be used for creating/deriving alert levels for real-world early warning systems^9,54^ and for numerical outputs capable of following the ground truth or actual malaria cases closely, thus allowing for inferred understanding of the variance which environmental forcing has on malaria cases.

The Transformer model indicates a higher predictive ability, with better accuracy especially when using multiple other climate variables as predictors (Fig. 7) in addition to rainfall and temperature, however it was still able to outperform the statistical and XGBOOST model even when only these two variables were used(Appendix 2 & 6). Despite this, there is still value in leveraging both frameworks^33^. Statistical models can help determine causality and highlight which climate variables or predictors are of value to the deep learning model, while also providing short-term forecasts to verify the DL model predictions^34^. However, explainability is an aspect that is also possible with these new Transformer models and should be explored in future studies^21^. Having converted the numerical case data to alert thresholds of three classes (low, medium, high), we have tested and evaluated the prediction of the models and find the Transformer performs better than the other models in predicting the high and low classes based on AUPRC. The AUROC echoes similar results. This gives us a robust picture of the threshold levels that can be reliably ascertained with the Transformer model. Therefore, in a real case scenario, the medium thresholds can be given more scrutiny and confirmed with the statistical or another model or combine multiple models to create a more representative and robust prediction output^43,55^. When Xu et al^42^. used AUROC scores to compare LSTM and Transformer models, the Transformer outperformed the LSTM consistently in addition to demonstrating higher accuracy metrics.

The fidelity and usefulness of a model’s predictions are necessary if it is to be applied in the real world^42^. The novel M-DELTA loss function appears to provide around 20% better accuracy than the base MSE loss function employed during model training (Appendix 2). Zerveas et al^40^. applied a dropout of 10% to prevent overfitting and promote generalisable predictions and found similarly that the Transformer outperformed the majority of existing best models (including XGBOOST and Neural Net models) on multiple different datasets. This is promising as it indicates that the Transformer may be similarly applied to other disease prediction tasks and datasets in the domain of climate related health outcomes and that the loss function developed for this study performs better than the established functions tested (Appendix 2). The use of the novel high temporal resolution malaria dataset allowed for a unique testing of climate-related disease prediction for a country in Southern Africa, where the application of deep learning predictive frameworks and particularly of the Transformer are not well understood^35,37^. The promising results from the Transformer now offer a practical solution to further incorporate more complex climate data, possible entomological data and other domain knowledge to improve and apply Transformer models to the task of malaria prediction in Africa.

## Conclusion

All models used only temperature and rainfall as predictors, however, the statistical model also used a log transform of actual malaria cases as an extra predictor, which gave the statistical model an unbalanced advantage in case prediction as observed in the lower accuracy when this advantage is removed. The Transformer is still able to outperform the statistical model across AUROC, AUPRC and regression metrics of evaluation (MAE, R^2^ and max error) despite this advantage and it is a trend that is becoming apparent when trying to model long-term, high temporal resolution outcomes based on complex data^33^. While the Transformer model still had problems when predicting medium case incidence, this can be attributed to a small dataset size and the post-prediction artificial classification method, as the regressive predictions were still highly accurate when compared to the numerical malaria case incidence. The alert levels derived from the numerical data still indicate utility, however for medium alert thresholds, using the Transformer data may require caution but this can be supported with other models in a real-world prediction framework. With larger datasets and addition of a larger array of climate parameters to explain unaccounted variability, these deep learning Transformers can be improved as seen with early tests^15,27^, which will provide valuable information in the effort against malaria prediction and mitigation.

### Supplementary Information

Supplementary Information.

## Data Availability

The climate data can be made available upon request and the Malaria case data may be shared to an extent after discussions with the stakeholders such as the Limpopo Health Department of South Africa due to private information disclosure and data ownership. Contact the corresponding author if data is required.
