# Supplementary material for: Utilizing a novel high-resolution malaria dataset for climate-informed predictions with a deep learning transformer model
Source: Sci Rep. 2023 Dec 28;13:23091. doi: 10.1038/s41598-023-50176-3 (PMC10754862; doi:10.1038/s41598-023-50176-3)
Supplement: Supplementary file 1 — Supplementary Information. [file 41598_2023_50176_MOESM1_ESM.docx]

**Appendix 1: Model Architecture**


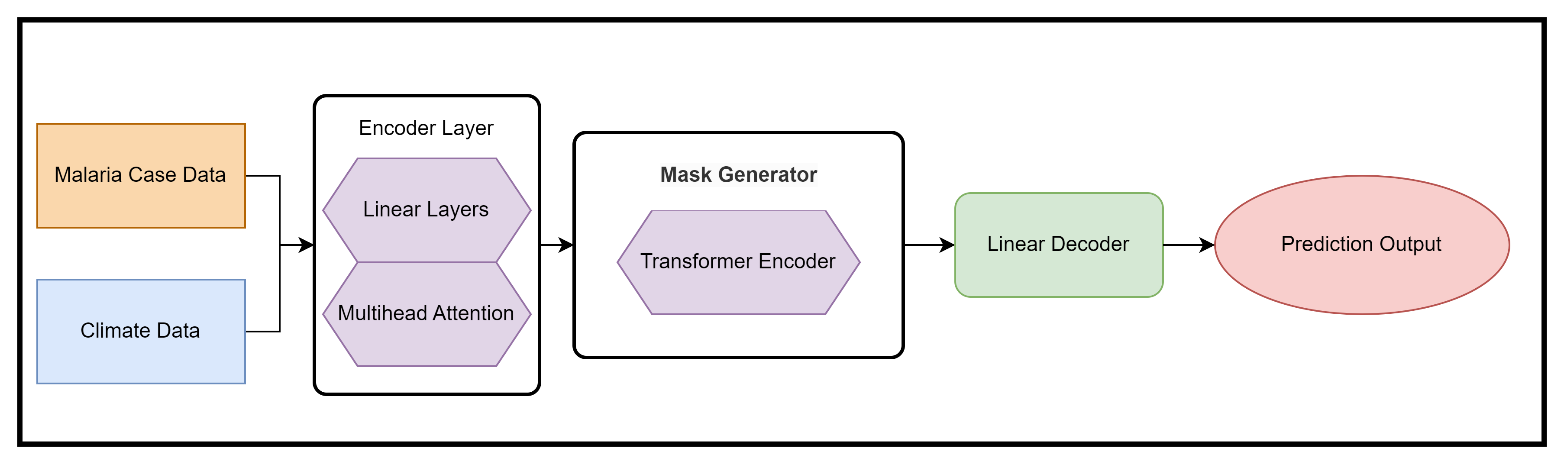


The simplified diagram of the transformer block used to train and predict malaria cases using climate and malaria data and a decoder-like architecture.


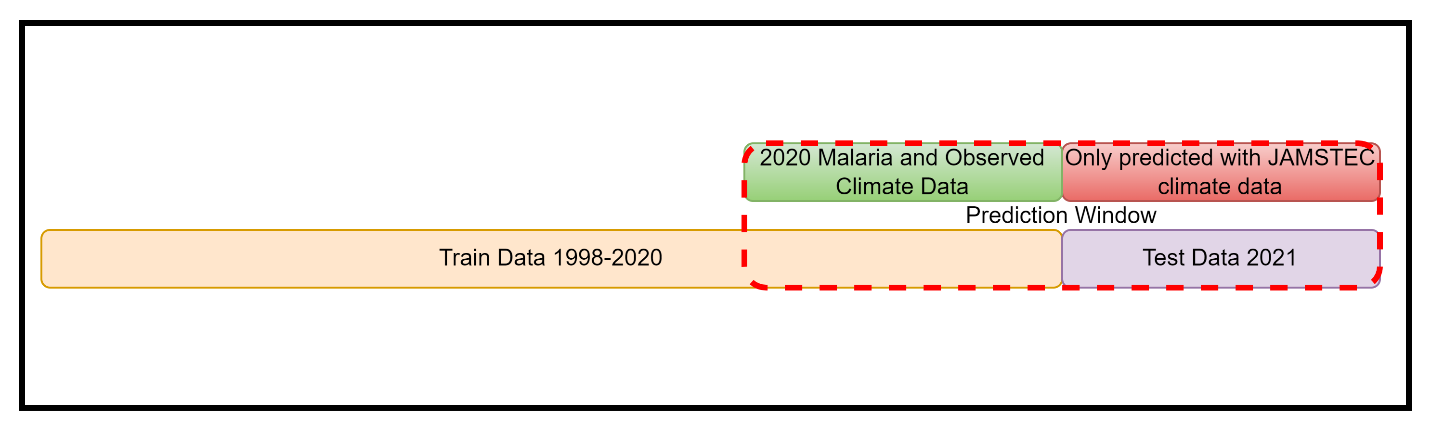


Supplementary explanation of figure 4c for Transformer prediction output. The model predicts two years ahead, however the first year is using the malaria data and observed climate data for the autoregressive prediction by using the past value to predict a step ahead and repeating until one year of predictions are attained. A mask is applied to prevent the model using the future values. This helps prime the model by giving it contextual information from the previous year so now it can predict with no malaria data for the 2021 period using only climate data derived from global climate model predictions. This gives us one year of real-world application predictions.

We employ a specialized Transformer architecture tailored for time-series forecasting. While the architecture uses components commonly found in a Transformer's encoder, it is applied in a "decoder-like" fashion, making it a "decoder-only" Transformer in the context of time-series prediction.

**Components**

- **Input Data**: The malaria and climate data used to train the model and make predictions.
- **Encoder Layer**: Comprises multi-head self-attention and feed-forward neural networks, with an attention head size of 7 and feature size of *d*_model_​=7.
- **Transformer Encoder**: Consists of multiple stacked encoder layers (default of 3 layers), each utilizing the aforementioned encoder layer structure.
- **Linear Decoder**: A fully connected linear layer that maps the *d*_model_​=7 feature size to a single output value, serving as the final prediction layer.
- **Prediction Output**: The numerical value prediction representing malaria case counts.

**Additional Mechanisms**

- **Masking(Mask Generator)**: A square subsequent mask is applied during the self-attention calculations to ensure that future information is not used in the prediction of the current or next value in the sequence.
- **Dropout**: A dropout rate of 0.2 is used for regularization. This parameter is used for overfitting prevention.

The architecture aims to leverage the parallelization and long-distance dependencies capturing advantages of the Transformer model to address specific challenges in time-series forecasting such as the importance of order and sequence length. The model concept is adapted from the GPT-2 architecture (See Radford *et al*.^52^). In the context of time-series forecasting, the architecture functions in a "decoder-like" fashion despite using Transformer encoder components. Important features that align it with the role of a decoder include the use of masked self-attention to prevent leakage of future information to the model when predicting/training, one-step-ahead predictions based on all available true values, and a direct mapping of the processed sequence to an output value via a linear layer. These characteristics together promote the generation of future time-series values from past data, mimicking the role traditionally played by a decoder in sequence-to-sequence models.

**Appendix 2: Model Parameters**

Model parameters tested during training and model development phase. Epochs, k, batch size, frequency, training length, forecast window, optimizer, loss function, weight initialization are adjusted variably until the model accuracy can be improved.

**Appendix 3**

A snapshot of the model during training. At epoch 0 the model is untrained, but by epoch 10K, the model can predict to match the observed malaria cases much more accurately.


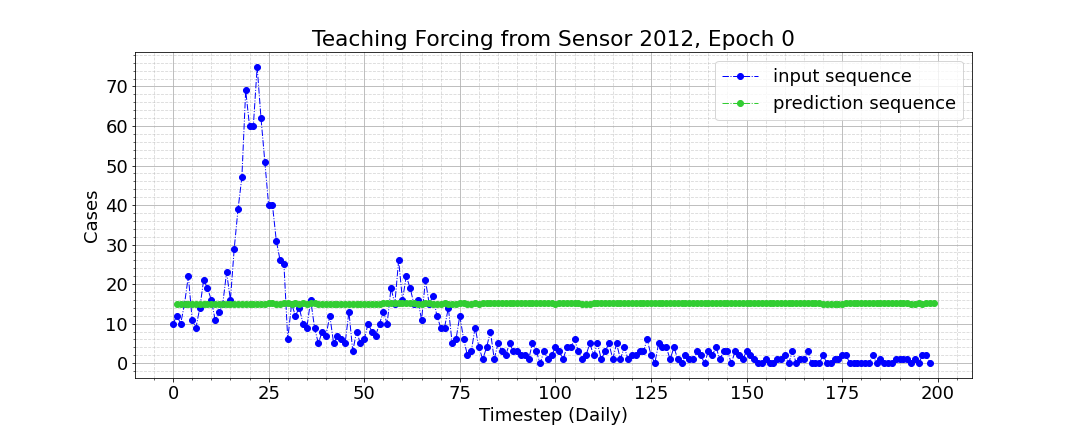

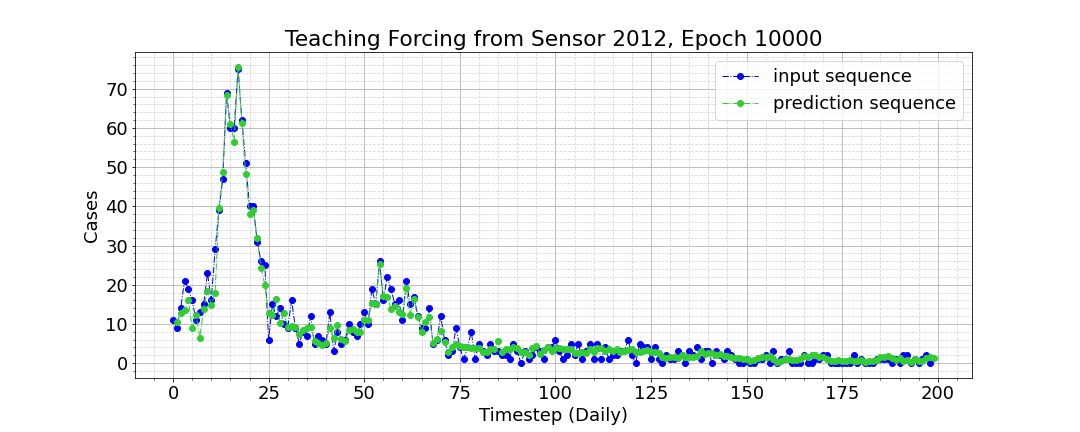


| **Index** | **Name** | **Type** | **Params** |
| --- | --- | --- | --- |
| 0 | loss | QuantileLoss | 0 |
| 1 | logging_metrics | ModuleList | 0 |
| 2 | input_embeddings | MultiEmbedding | 0 |
| 3 | prescalers | ModuleDict | 256 |
| 4 | static_variable_selection | VariableSelectionNetwork | 3.0 K |
| 5 | encoder_variable_selection | VariableSelectionNetwork | 7.4 K |
| 6 | decoder_variable_selection | VariableSelectionNetwork | 2.4 K |
| 7 | static_context_variable_selection | GatedResidualNetwork | 1.1 K |
| 8 | static_context_initial_hidden_lstm | GatedResidualNetwork | 1.1 K |
| 9 | static_context_initial_cell_lstm | GatedResidualNetwork | 1.1 K |
| 10 | static_context_enrichment | GatedResidualNetwork | 1.1 K |
| 11 | lstm_encoder | LSTM | 2.2 K |
| 12 | lstm_decoder | LSTM | 2.2 K |
| 13 | post_lstm_gate_encoder | GatedLinearUnit | 544 |
| 14 | post_lstm_add_norm_encoder | AddNorm | 32 |
| 15 | static_enrichment | GatedResidualNetwork | 1.4 K |
| 16 | multihead_attn | InterpretableMultiHeadAttention | 808 |
| 17 | post_attn_gate_norm | GateAddNorm | 576 |
| 18 | pos_wise_ff | GatedResidualNetwork | 1.1 K |
| 19 | pre_output_gate_norm | GateAddNorm | 576 |
| 20 | output_layer | Linear | 119 |

**Appendix 4: Model Parameters and input structure**

**Appendix 5: Different Temporal resolution Predictions**

**
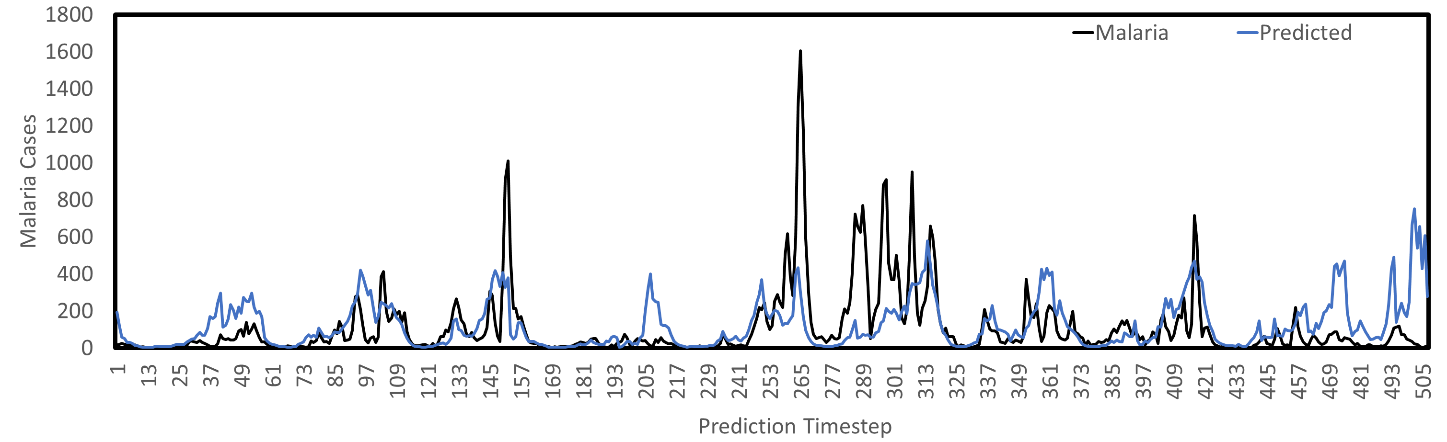
**

Daily prediction for statistical model.


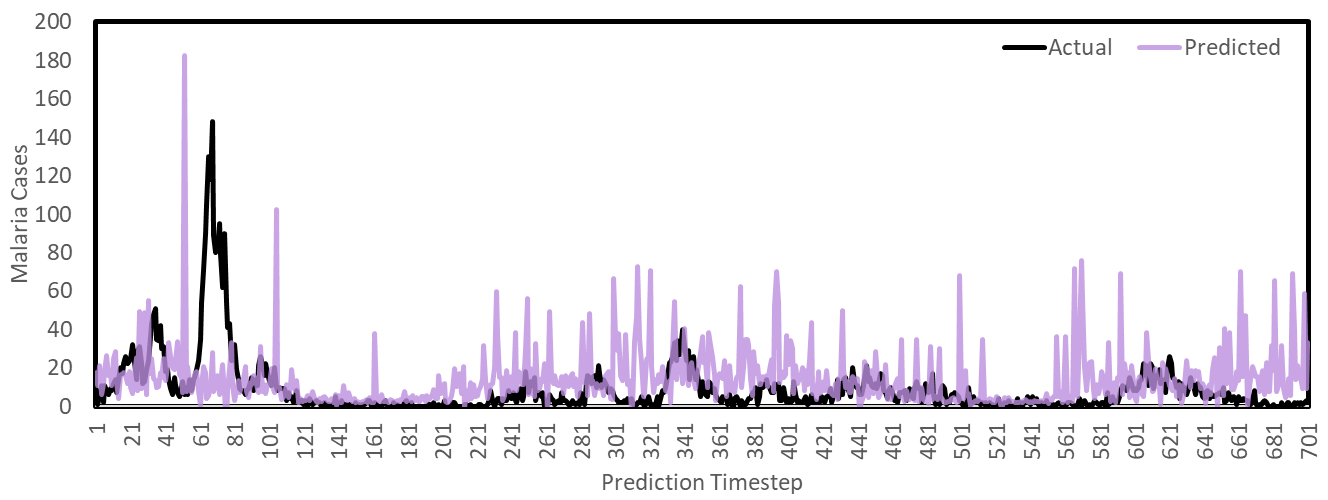


Daily prediction for XGBOOST model.


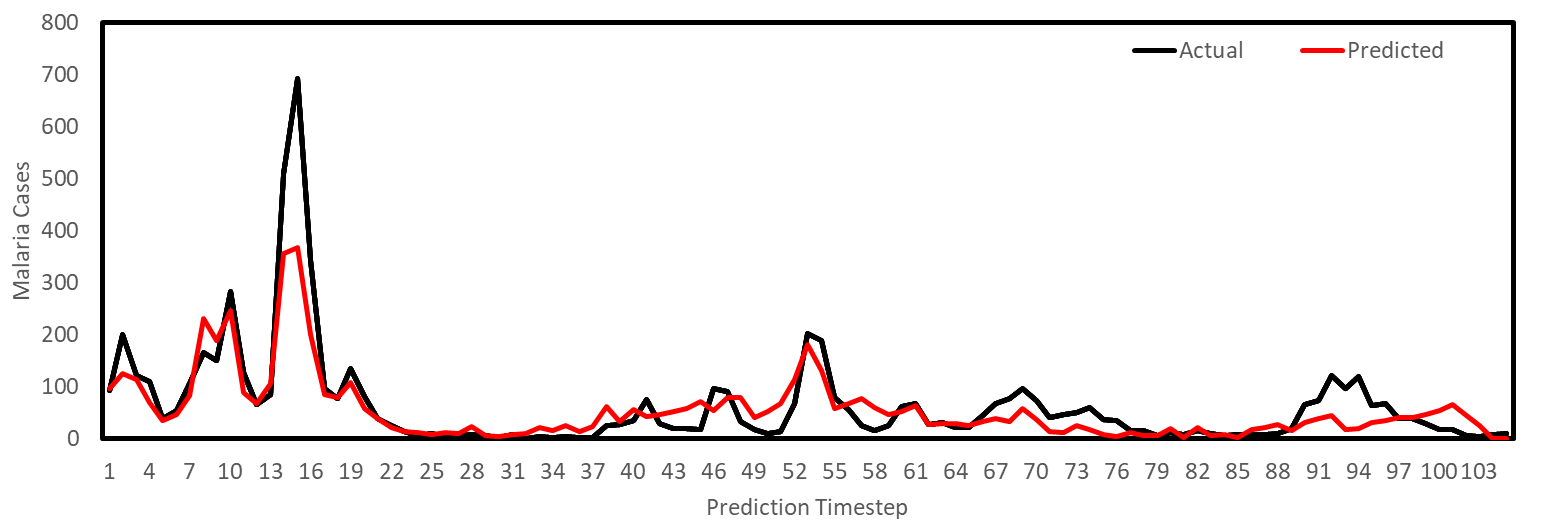


Weekly downscaled prediction from the deep learning transformer model.

**Appendix 6: Correlation of Predictions with Ground Truth (Actual) Values**


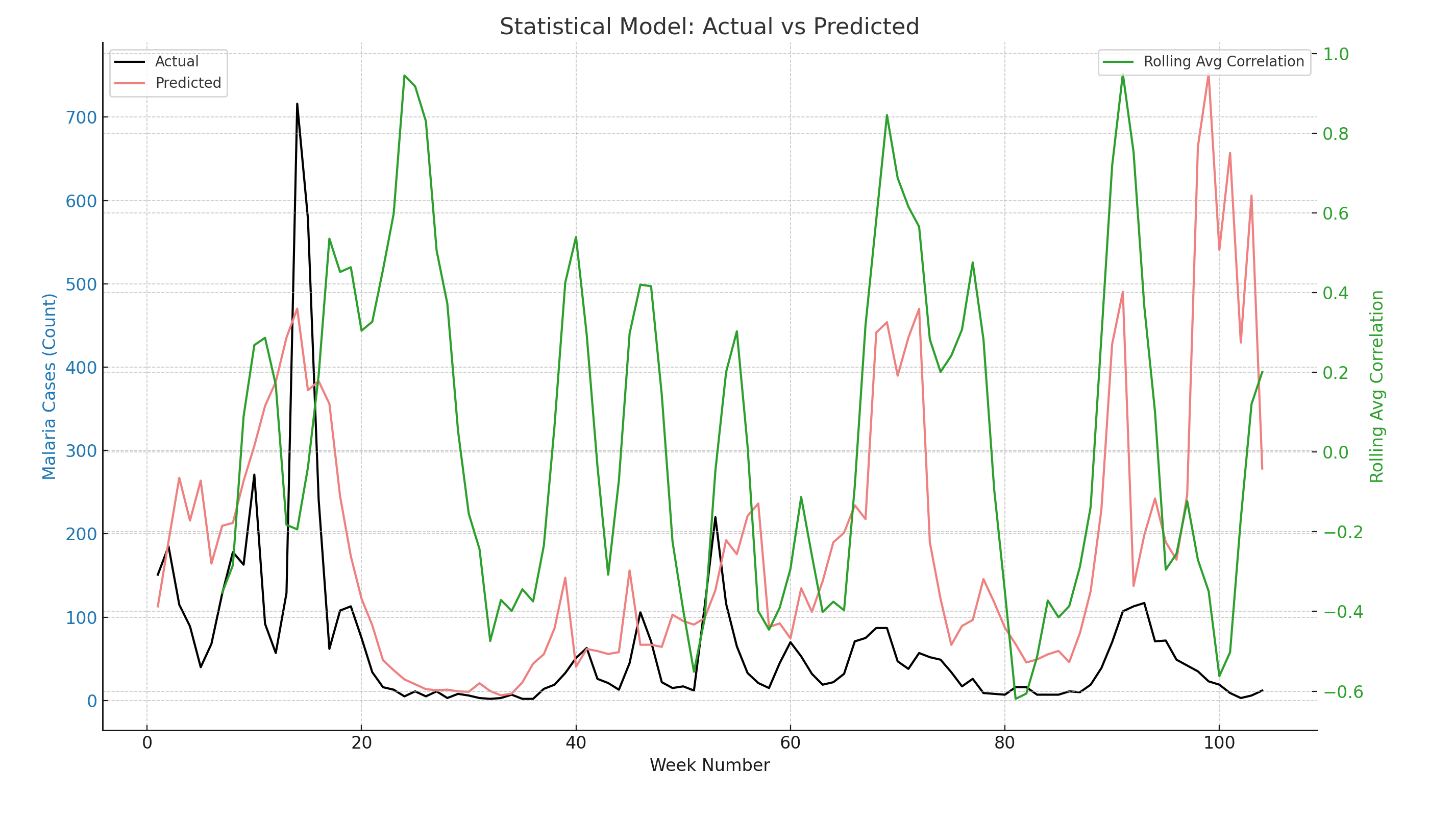


Predictions for Statistical Model and 10 week rolling window correlation for predictions.


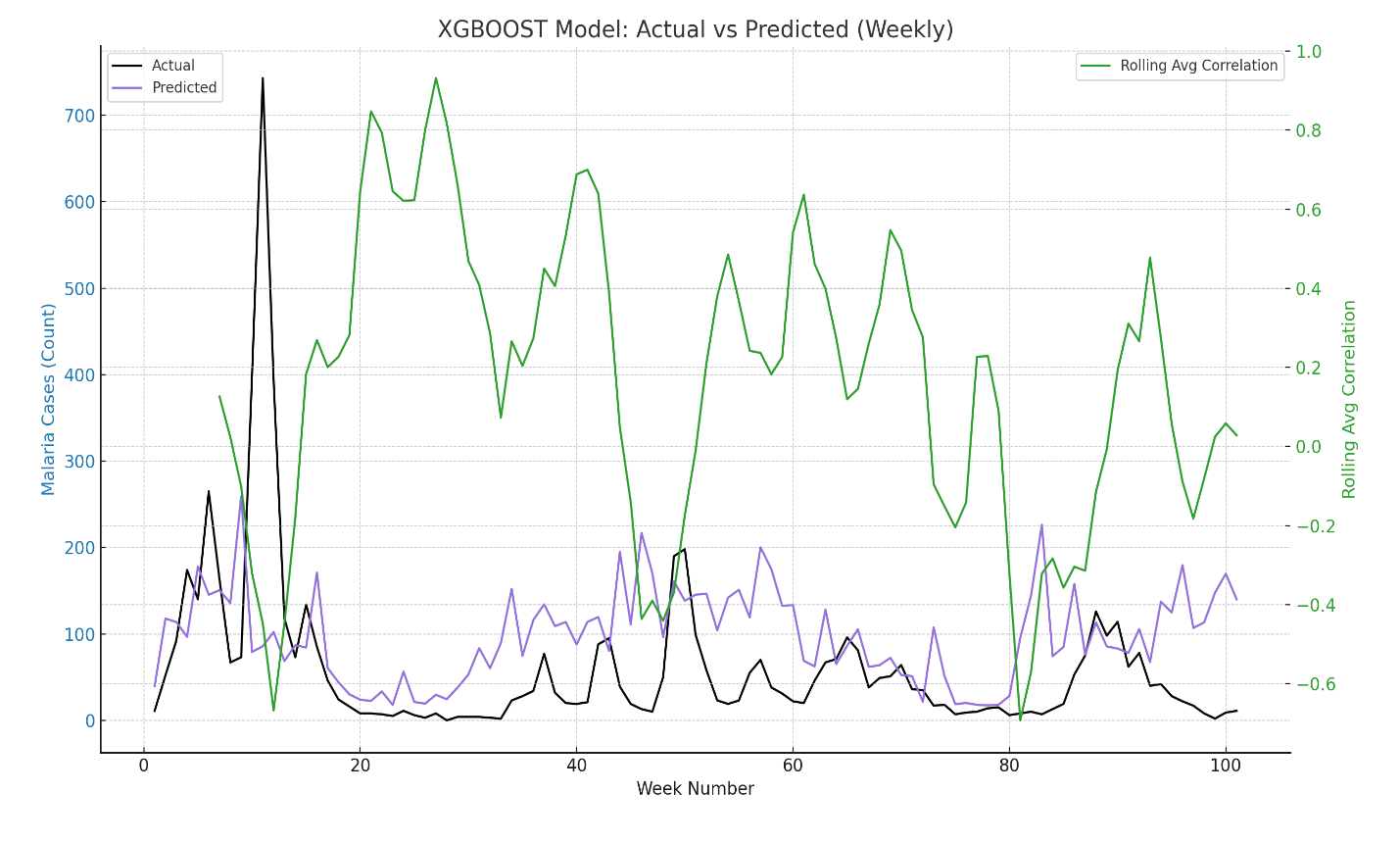


Predictions for XGBOOST Model and 10 week rolling window correlation for predictions.

| Metrics | Statistical | XGBOOST (ML) | Transformer (DL) |
| --- | --- | --- | --- |
| Accuracy | 55-79% | 43% | 98% |
| Prediction Resolution | Weekly | Daily/Weekly | Daily |
| Timesteps ahead | 16 Weeks | 2 years (+700 days) | 200-365 days |
| Reliability based on AUC | 40% | 60% | 80% |
| Training Time | Immediate | Immediate | 2 weeks |


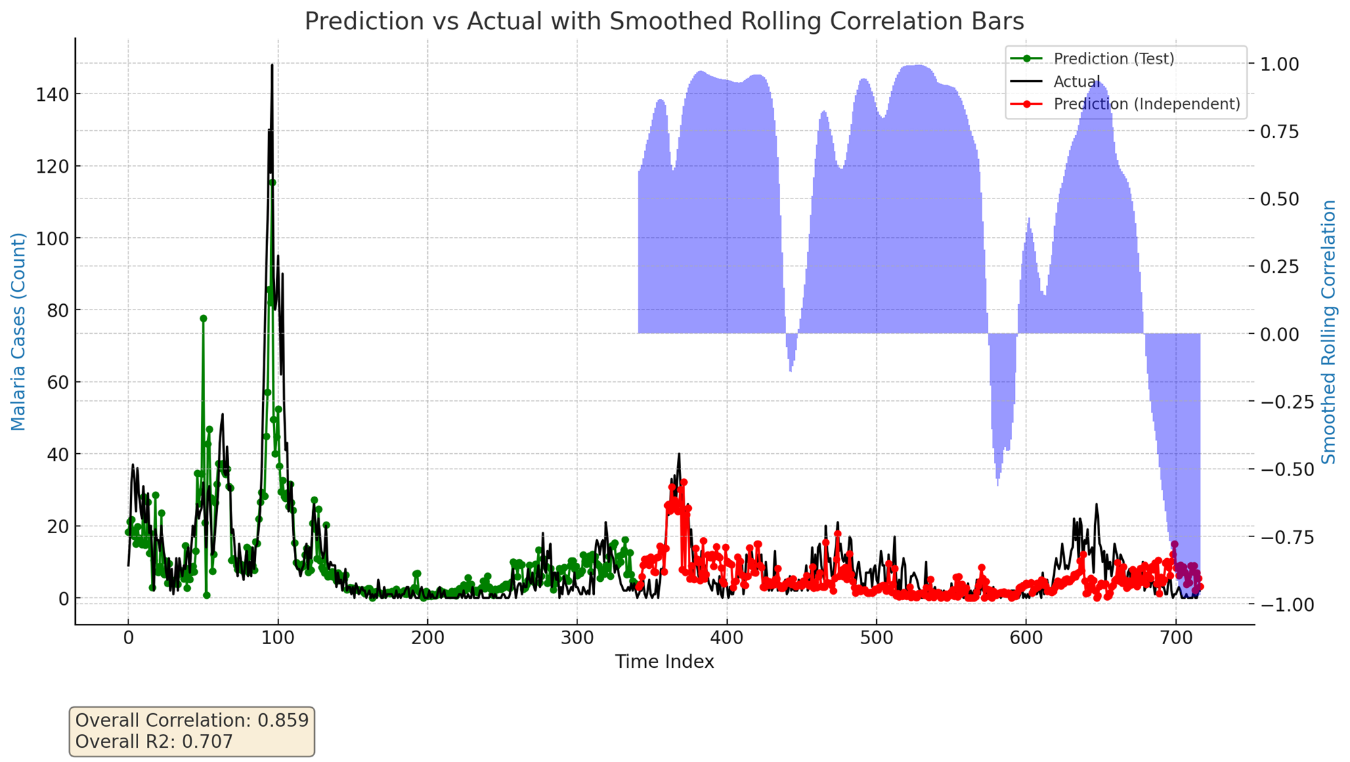


Predictions for Transformer Model and 50 day rolling window correlation for predictions.

**Appendix 6: Summary of results**
